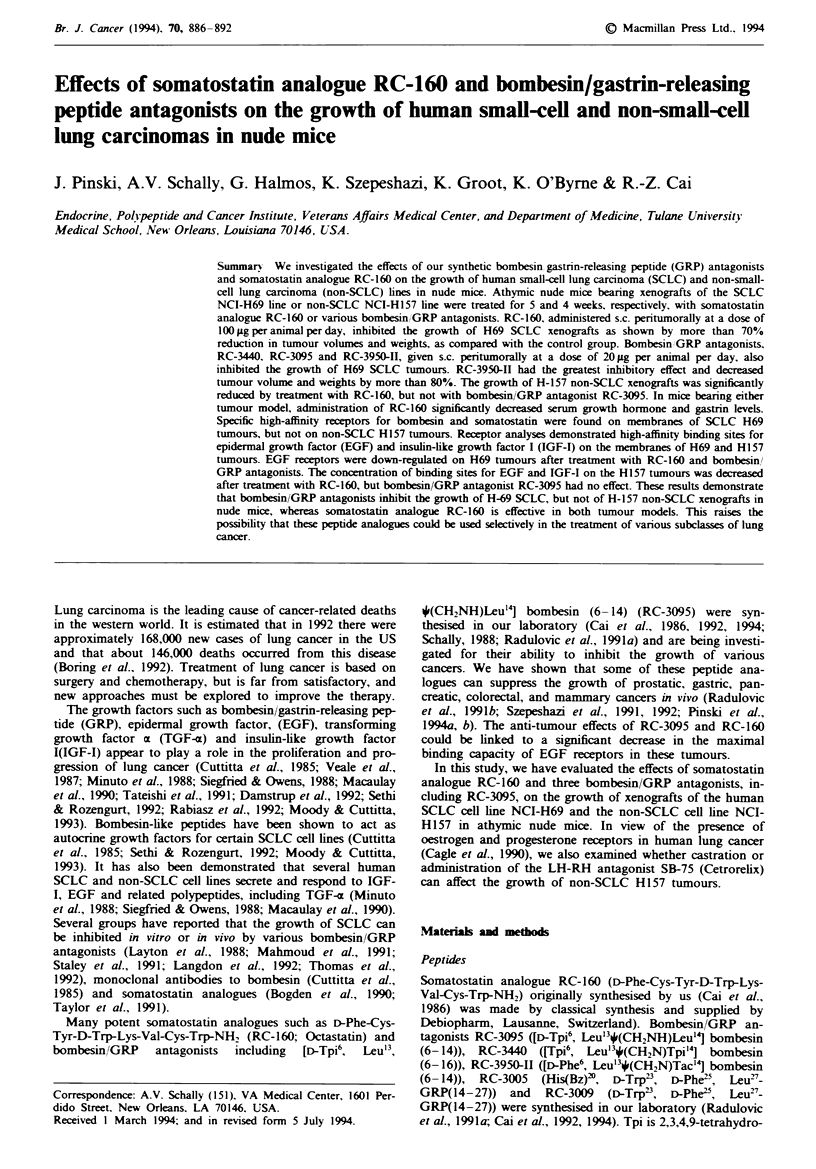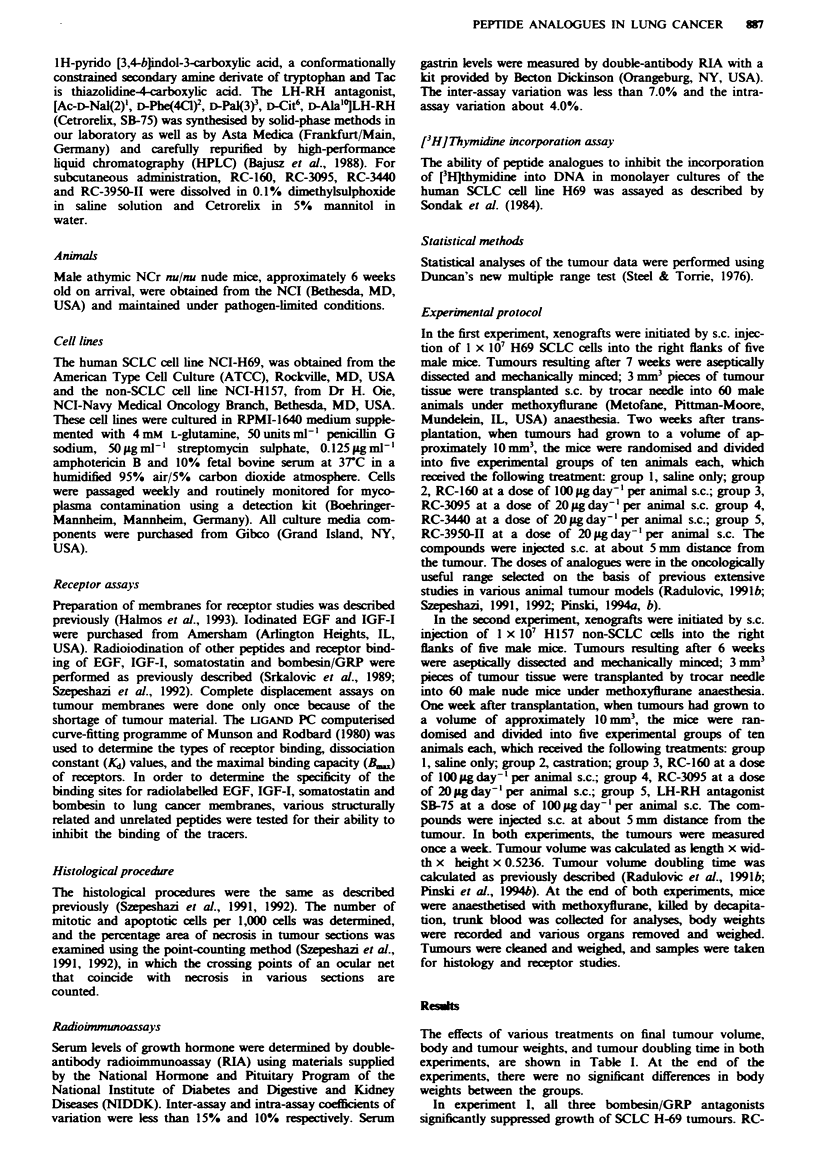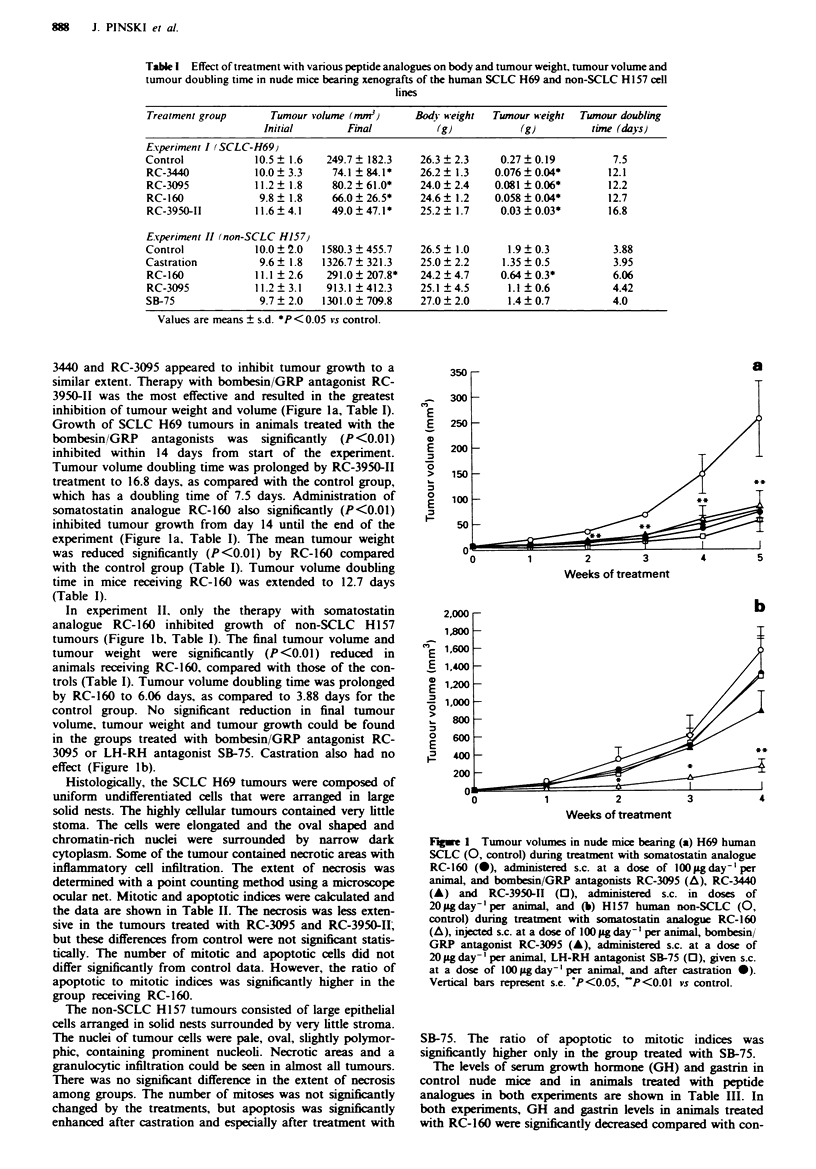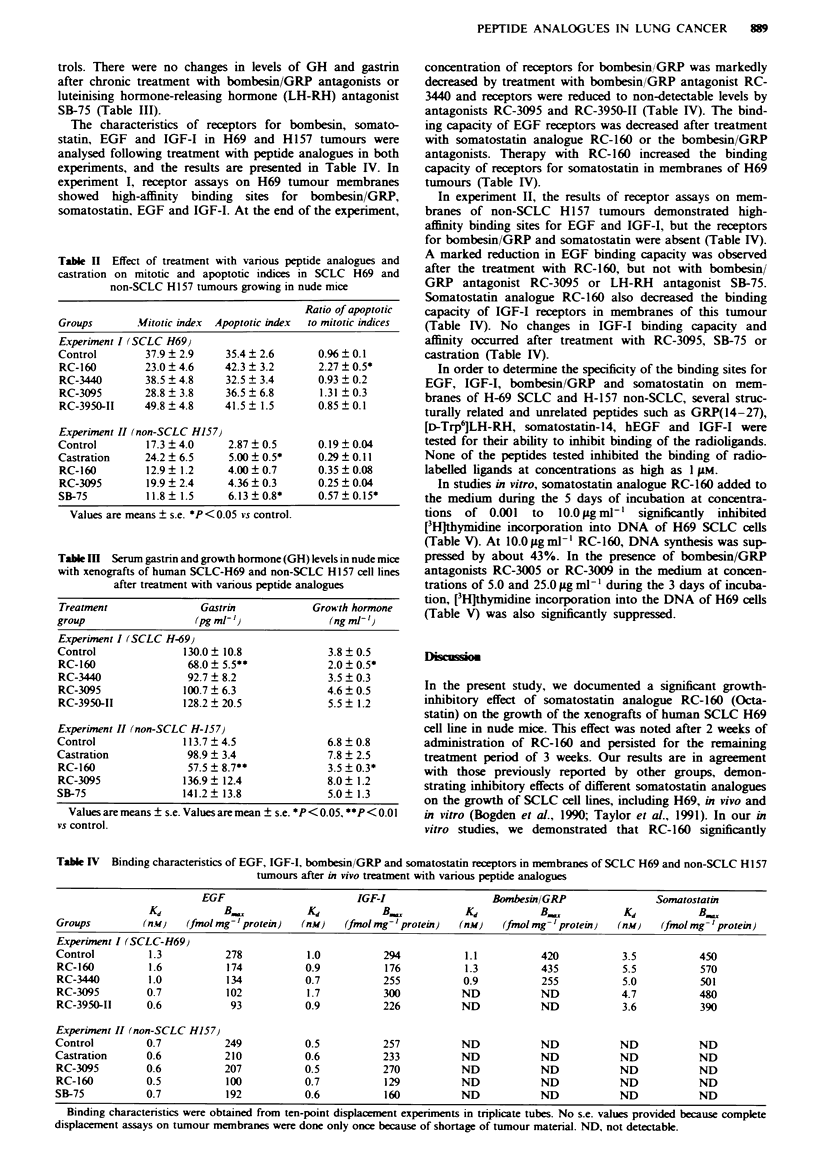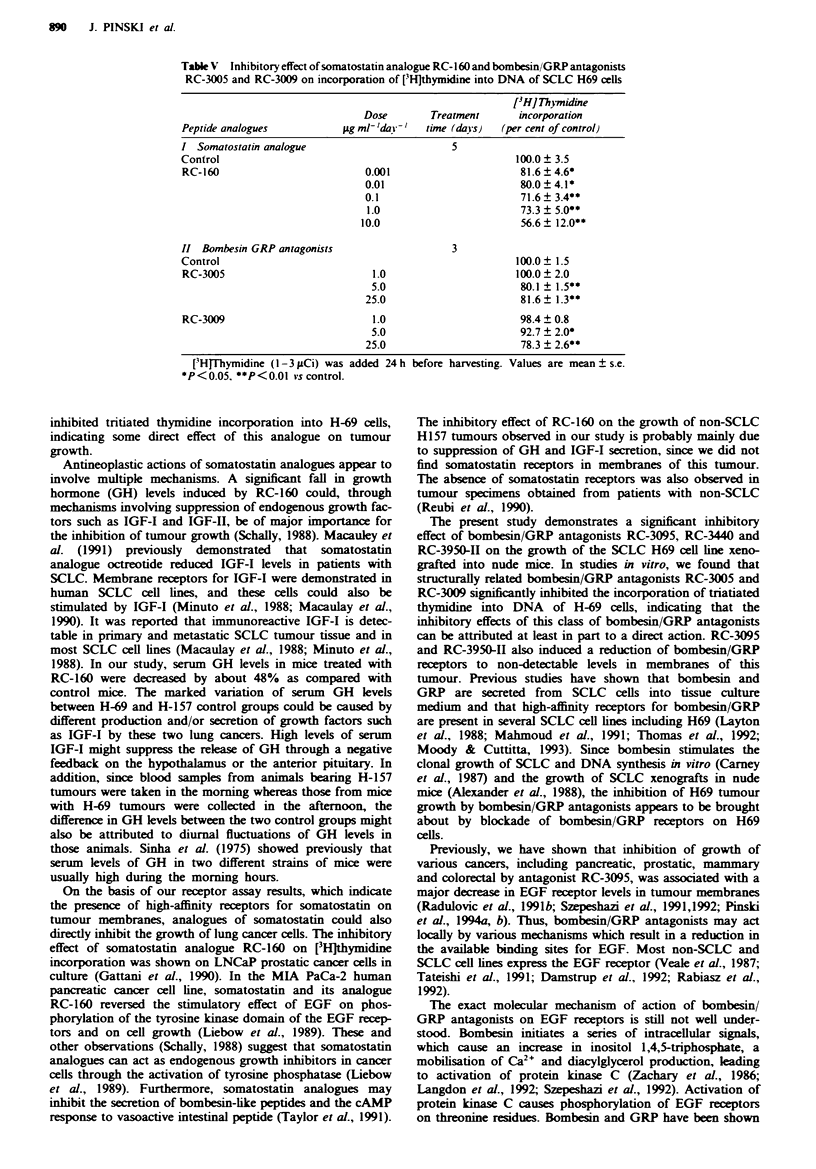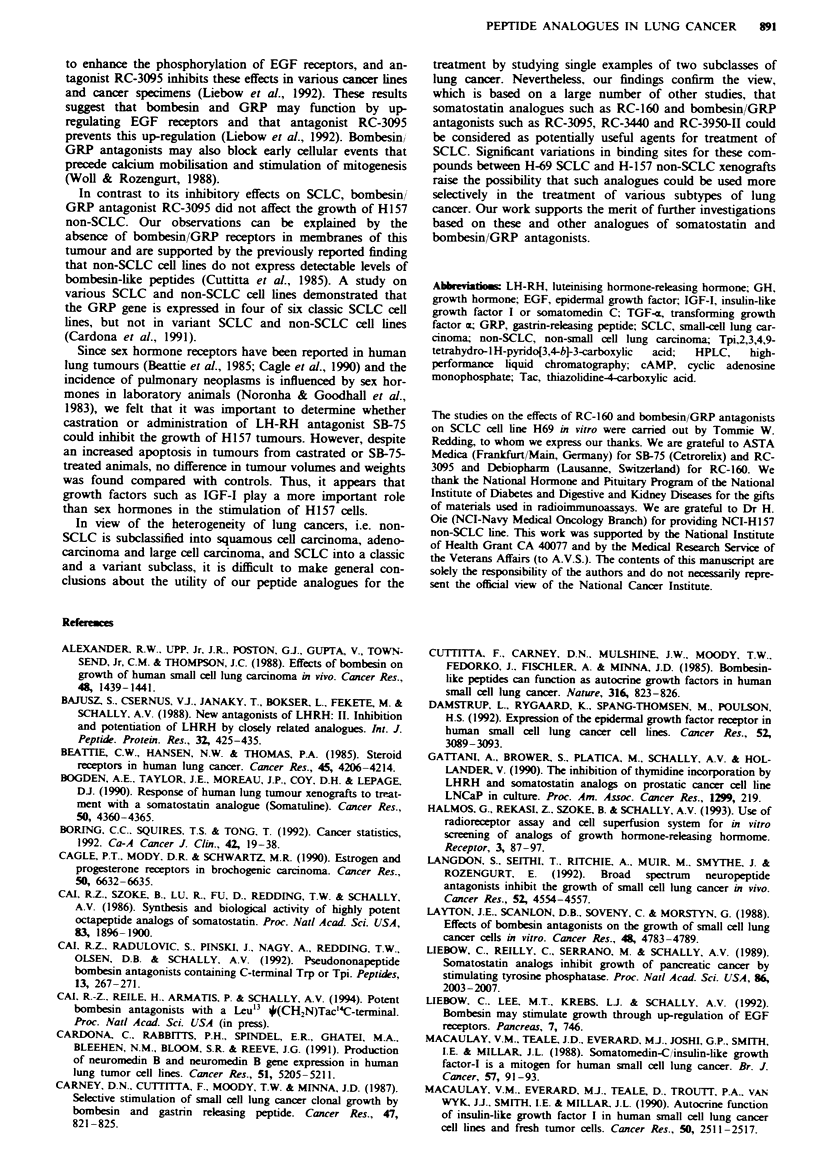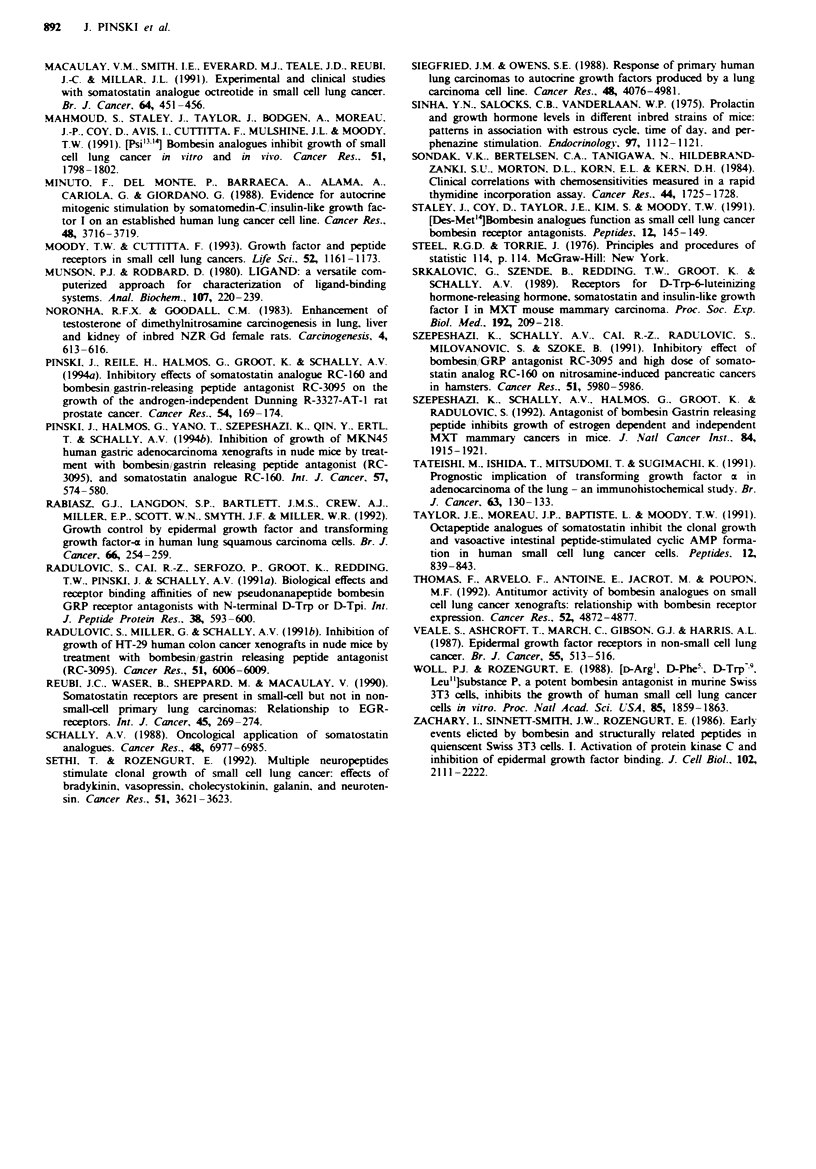# Effects of somatostatin analogue RC-160 and bombesin/gastrin-releasing peptide antagonists on the growth of human small-cell and non-small-cell lung carcinomas in nude mice.

**DOI:** 10.1038/bjc.1994.415

**Published:** 1994-11

**Authors:** J. Pinski, A. V. Schally, G. Halmos, K. Szepeshazi, K. Groot, K. O'Byrne, R. Z. Cai

**Affiliations:** Endocrine, Polypeptide and Cancer Institute, Veterans Affairs Medical Center, New Orleans, Louisiana.

## Abstract

We investigated the effects of our synthetic bombesin/gastrin-releasing peptide (GRP) antagonists and somatostatin analogue RC-160 on the growth of human small-cell lung carcinoma (SCLC) and non-small-cell lung carcinoma (non-SCLC) lines in nude mice. Athymic nude mice bearing xenografts of the SCLC NCl-H69 line or non-SCLC NCl-H157 line were treated for 5 and 4 weeks, respectively, with somatostatin analogue RC-160 or various bombesin/GRP antagonists. RC-160, administered s.c. peritumorally at a dose of 100 micrograms per animal per day, inhibited the growth of H69 SCLC xenografts as shown by more than 70% reduction in tumour volumes and weights, as compared with the control group. Bombesin/GRP antagonists, RC-3440, RC-3095 and RC-3950-II, given s.c. peritumorally at a dose of 20 micrograms per animal per day, also inhibited the growth of H69 SCLC tumours. RC-3950-II had the greatest inhibitory effect and decreased tumour volume and weights by more than 80%. The growth of H-157 non-SCLC xenografts was significantly reduced by treatment with RC-160, but not with bombesin/GRP antagonist RC-3095. In mice bearing either tumour model, administration of RC-160 significantly decreased serum growth hormone and gastrin levels. Specific high-affinity receptors for bombesin and somatostatin were found on membranes of SCLC H69 tumours, but not on non-SCLC H157 tumours. Receptor analyses demonstrated high-affinity binding sites for epidermal growth factor (EGF) and insulin-like growth factor I (IGF-I) on the membranes of H69 and H157 tumours. EGF receptors were down-regulated on H69 tumours after treatment with RC-160 and bombesin/GRP antagonists. The concentration of binding sites for EGF and IGF-I on the H157 tumours was decreased after treatment with RC-160, but bombesin/GRP antagonist RC-3095 had no effect. These results demonstrate that bombesin/GRP antagonists inhibit the growth of H-69 SCLC, but not of H-157 non-SCLC xenografts in nude mice, whereas somatostatin analogue RC-160 is effective in both tumour models. This raises the possibility that these peptide analogues could be used selectively in the treatment of various subclasses of lung cancer.


Br. J. Cancer (1994), 70, 886-892 © Macmillan Press Ltd., 1994

### Effects of somatostatin analogue RC-160 and bombesin/gastrin-releasing peptide antagonists on the growth of human small-cell and non-small-cell lung carcinomas in nude mice

J. Pinski, A.V. Schally, G. Halmos, K. Szepeshazi, K. Groot, K. O'Byrne & R.-Z. Cai

*Endocrine, Polypeptide and Cancer Institute, Veterans Affairs Medical Center, and Department of Medicine, Tulane University Medical School, New Orleans, Louisiana 70146, USA.*

**Summary** We investigated the effects of our synthetic bombesin gastrin-releasing peptide (GRP) antagonists and somatostatin analogue RC-160 on the growth of human small-cell lung carcinoma (SCLC) and non-small-cell lung carcinoma (non-SCLC) lines in nude mice. Athymic nude mice bearing xenografts of the SCLC NCI-H69 line or non-SCLC NCI-H157 line were treated for 5 and 4 weeks, respectively, with somatostatin analogue RC-160 or various bombesin/GRP antagonists. RC-160, administered s.c. peritumorally at a dose of 100 μg per animal per day, inhibited the growth of H69 SCLC xenografts as shown by more than 70% reduction in tumour volumes and weights, as compared with the control group. Bombesin/GRP antagonists, RC-3440, RC-3095 and RC-3950-II, given s.c. peritumorally at a dose of 20 μg per animal per day, also inhibited the growth of H69 SCLC tumours. RC-3950-II had the greatest inhibitory effect and decreased tumour volume and weights by more than 80%. The growth of H-157 non-SCLC xenografts was significantly reduced by treatment with RC-160, but not with bombesin/GRP antagonist RC-3095. In mice bearing either tumour model, administration of RC-160 significantly decreased serum growth hormone and gastrin levels. Specific high-affinity receptors for bombesin and somatostatin were found on membranes of SCLC H69 tumours, but not on non-SCLC H157 tumours. Receptor analyses demonstrated high-affinity binding sites for epidermal growth factor (EGF) and insulin-like growth factor I (IGF-I) on the membranes of H69 and H157 tumours. EGF receptors were down-regulated on H69 tumours after treatment with RC-160 and bombesin/GRP antagonists. The concentration of binding sites for EGF and IGF-I on the H157 tumours was decreased after treatment with RC-160, but bombesin/GRP antagonist RC-3095 had no effect. These results demonstrate that bombesin/GRP antagonists inhibit the growth of H-69 SCLC, but not of H-157 non-SCLC xenografts in nude mice, whereas somatostatin analogue RC-160 is effective in both tumour models. This raises the possibility that these peptide analogues could be used selectively in the treatment of various subclasses of lung cancer.

Lung carcinoma is the leading cause of cancer-related deaths in the western world. It is estimated that in 1992 there were approximately 168,000 new cases of lung cancer in the US and that about 146,000 deaths occurred from this disease (Boring *et al.*, 1992). Treatment of lung cancer is based on surgery and chemotherapy, but is far from satisfactory, and new approaches must be explored to improve the therapy.

The growth factors such as bombesin/gastrin-releasing peptide (GRP), epidermal growth factor, (EGF), transforming growth factor α (TGF-α) and insulin-like growth factor I(IGF-I) appear to play a role in the proliferation and progression of lung cancer (Cuttitta *et al.*, 1985; Veale *et al.*, 1987; Minuto *et al.*, 1988; Siegfried & Owens, 1988; Macaulay *et al.*, 1990; Tateishi *et al.*, 1991; Damstrup *et al.*, 1992; Sethi & Rozengurt, 1992; Rabiasz *et al.*, 1992; Moody & Cuttitta, 1993). Bombesin-like peptides have been shown to act as autocrine growth factors for certain SCLC cell lines (Cuttitta *et al.*, 1985; Sethi & Rozengurt, 1992; Moody & Cuttitta, 1993). It has also been demonstrated that several human SCLC and non-SCLC cell lines secrete and respond to IGF-I, EGF and related polypeptides, including TGF-α (Minuto *et al.*, 1988; Siegfried & Owens, 1988; Macaulay *et al.*, 1990). Several groups have reported that the growth of SCLC can be inhibited *in vitro* or *in vivo* by various bombesin/GRP antagonists (Layton *et al.*, 1988; Mahmoud *et al.*, 1991; Staley *et al.*, 1991; Langdon *et al.*, 1992; Thomas *et al.*, 1992), monoclonal antibodies to bombesin (Cuttitta *et al.*, 1985) and somatostatin analogues (Bogden *et al.*, 1990; Taylor *et al.*, 1991).

Many potent somatostatin analogues such as D-Phe-Cys-Tyr-D-Trp-Lys-Val-Cys-Trp-$NH_2$ (RC-160; Octastatin) and bombesin/GRP antagonists including [D-Tpi$^6$, Leu$^{13}$-$\psi(CH_2NH)$Leu$^{14}$] bombesin (6-14) (RC-3095) were synthesised in our laboratory (Cai *et al.*, 1986, 1992, 1994; Schally, 1988; Radulovic *et al.*, 1991*a*) and are being investigated for their ability to inhibit the growth of various cancers. We have shown that some of these peptide analogues can suppress the growth of prostatic, gastric, pancreatic, colorectal, and mammary cancers *in vivo* (Radulovic *et al.*, 1991*b*; Szepeshazi *et al.*, 1991, 1992; Pinski *et al.*, 1994*a*, *b*). The anti-tumour effects of RC-3095 and RC-160 could be linked to a significant decrease in the maximal binding capacity of EGF receptors in these tumours.

In this study, we have evaluated the effects of somatostatin analogue RC-160 and three bombesin/GRP antagonists, including RC-3095, on the growth of xenografts of the human SCLC cell line NCI-H69 and the non-SCLC cell line NCI-H157 in athymic nude mice. In view of the presence of oestrogen and progesterone receptors in human lung cancer (Cagle *et al.*, 1990), we also examined whether castration or administration of the LH-RH antagonist SB-75 (Cetrorelix) can affect the growth of non-SCLC H157 tumours.

### Materials and methods

### Peptides

Somatostatin analogue RC-160 (D-Phe-Cys-Tyr-D-Trp-Lys-Val-Cys-Trp-$NH_2$) originally synthesised by us (Cai *et al.*, 1986) was made by classical synthesis and supplied by Debiopharm, Lausanne, Switzerland). Bombesin/GRP antagonists RC-3095 ([D-Tpi$^6$, Leu$^{13}\psi(CH_2NH)$Leu$^{14}$] bombesin (6-14)), RC-3440 ([Tpi$^6$, Leu$^{13}\psi(CH_2N)$Tpi$^{14}$] bombesin (6-16)), RC-3950-II ([D-Phe$^6$, Leu$^{13}\psi(CH_2N)$Tac$^{14}$] bombesin (6-14)), RC-3005 (His(Bz)$^{20}$, D-Trp$^{23}$, D-Phe$^{25}$, Leu$^{27}$-GRP(14-27)) and RC-3009 (D-Trp$^{23}$, D-Phe$^{25}$, Leu$^{27}$-GRP(14-27)) were synthesised in our laboratory (Radulovic *et al.*, 1991*a*; Cai *et al.*, 1992, 1994). Tpi is 2,3,4,9-tetrahydro-

Correspondence: A.V. Schally (151), VA Medical Center, 1601 Perdido Street, New Orleans, LA 70146, USA.
Received 1 March 1994; and in revised form 5 July 1994.

1H-pyrido [3,4-*b*]indol-3-carboxylic acid, a conformationally constrained secondary amine derivate of tryptophan and Tac is thiazolidine-4-carboxylic acid. The LH-RH antagonist, [Ac-D-Nal(2)$^1$, D-Phe(4Cl)$^2$, D-Pal(3)$^3$, D-Cit$^6$, D-Ala$^{10}$]LH-RH (Cetrorelix, SB-75) was synthesised by solid-phase methods in our laboratory as well as by Asta Medica (Frankfurt/Main, Germany) and carefully repurified by high-performance liquid chromatography (HPLC) (Bajusz *et al.*, 1988). For subcutaneous administration, RC-160, RC-3095, RC-3440 and RC-3950-II were dissolved in 0.1% dimethylsulphoxide in saline solution and Cetrorelix in 5% mannitol in water.

*Animals*

Male athymic NCr *nu/nu* nude mice, approximately 6 weeks old on arrival, were obtained from the NCI (Bethesda, MD, USA) and maintained under pathogen-limited conditions.

*Cell lines*

The human SCLC cell line NCI-H69, was obtained from the American Type Cell Culture (ATCC), Rockville, MD, USA and the non-SCLC cell line NCI-H157, from Dr H. Oie, NCI-Navy Medical Oncology Branch, Bethesda, MD, USA. These cell lines were cultured in RPMI-1640 medium supplemented with 4 mM L-glutamine, 50 units ml$^{-1}$ penicillin G sodium, 50 μg ml$^{-1}$ streptomycin sulphate, 0.125 μg ml$^{-1}$ amphotericin B and 10% fetal bovine serum at 37°C in a humidified 95% air/5% carbon dioxide atmosphere. Cells were passaged weekly and routinely monitored for mycoplasma contamination using a detection kit (Boehringer-Mannheim, Mannheim, Germany). All culture media components were purchased from Gibco (Grand Island, NY, USA).

*Receptor assays*

Preparation of membranes for receptor studies was described previously (Halmos *et al.*, 1993). Iodinated EGF and IGF-I were purchased from Amersham (Arlington Heights, IL, USA). Radioiodination of other peptides and receptor binding of EGF, IGF-I, somatostatin and bombesin/GRP were performed as previously described (Srkalovic *et al.*, 1989; Szepeshazi *et al.*, 1992). Complete displacement assays on tumour membranes were done only once because of the shortage of tumour material. The LIGAND PC computerised curve-fitting programme of Munson and Rodbard (1980) was used to determine the types of receptor binding, dissociation constant ($K_d$) values, and the maximal binding capacity ($B_{max}$) of receptors. In order to determine the specificity of the binding sites for radiolabelled EGF, IGF-I, somatostatin and bombesin to lung cancer membranes, various structurally related and unrelated peptides were tested for their ability to inhibit the binding of the tracers.

*Histological procedure*

The histological procedures were the same as described previously (Szepeshazi *et al.*, 1991, 1992). The number of mitotic and apoptotic cells per 1,000 cells was determined, and the percentage area of necrosis in tumour sections was examined using the point-counting method (Szepeshazi *et al.*, 1991, 1992), in which the crossing points of an ocular net that coincide with necrosis in various sections are counted.

*Radioimmunoassays*

Serum levels of growth hormone were determined by double-antibody radioimmunoassay (RIA) using materials supplied by the National Hormone and Pituitary Program of the National Institute of Diabetes and Digestive and Kidney Diseases (NIDDK). Inter-assay and intra-assay coefficients of variation were less than 15% and 10% respectively. Serum gastrin levels were measured by double-antibody RIA with a kit provided by Becton Dickinson (Orangeburg, NY, USA). The inter-assay variation was less than 7.0% and the intra-assay variation about 4.0%.

*[$^3$H]Thymidine incorporation assay*

The ability of peptide analogues to inhibit the incorporation of [$^3$H]thymidine into DNA in monolayer cultures of the human SCLC cell line H69 was assayed as described by Sondak *et al.* (1984).

*Statistical methods*

Statistical analyses of the tumour data were performed using Duncan's new multiple range test (Steel & Torrie, 1976).

*Experimental protocol*

In the first experiment, xenografts were initiated by s.c. injection of 1 × 10$^7$ H69 SCLC cells into the right flanks of five male mice. Tumours resulting after 7 weeks were aseptically dissected and mechanically minced; 3 mm$^3$ pieces of tumour tissue were transplanted s.c. by trocar needle into 60 male animals under methoxyflurane (Metofane, Pittman-Moore, Mundelein, IL, USA) anaesthesia. Two weeks after transplantation, when tumours had grown to a volume of approximately 10 mm$^3$, the mice were randomised and divided into five experimental groups of ten animals each, which received the following treatment: group 1, saline only; group 2, RC-160 at a dose of 100 μg day$^{-1}$ per animal s.c.; group 3, RC-3095 at a dose of 20 μg day$^{-1}$ per animal s.c. group 4, RC-3440 at a dose of 20 μg day$^{-1}$ per animal s.c.; group 5, RC-3950-II at a dose of 20 μg day$^{-1}$ per animal s.c. The compounds were injected s.c. at about 5 mm distance from the tumour. The doses of analogues were in the oncologically useful range selected on the basis of previous extensive studies in various animal tumour models (Radulovic, 1991*b*; Szepeshazi, 1991, 1992; Pinski, 1994*a*, *b*).

In the second experiment, xenografts were initiated by s.c. injection of 1 × 10$^7$ H157 non-SCLC cells into the right flanks of five male mice. Tumours resulting after 6 weeks were aseptically dissected and mechanically minced; 3 mm$^3$ pieces of tumour tissue were transplanted by trocar needle into 60 male nude mice under methoxyflurane anaesthesia. One week after transplantation, when tumours had grown to a volume of approximately 10 mm$^3$, the mice were randomised and divided into five experimental groups of ten animals each, which received the following treatments: group 1, saline only; group 2, castration; group 3, RC-160 at a dose of 100 μg day$^{-1}$ per animal s.c.; group 4, RC-3095 at a dose of 20 μg day$^{-1}$ per animal s.c.; group 5, LH-RH antagonist SB-75 at a dose of 100 μg day$^{-1}$ per animal s.c. The compounds were injected s.c. at about 5 mm distance from the tumour. In both experiments, the tumours were measured once a week. Tumour volume was calculated as length × width × height × 0.5236. Tumour volume doubling time was calculated as previously described (Radulovic *et al.*, 1991*b*; Pinski *et al.*, 1994*b*). At the end of both experiments, mice were anaesthetised with methoxyflurane, killed by decapitation, trunk blood was collected for analyses, body weights were recorded and various organs removed and weighed. Tumours were cleaned and weighed, and samples were taken for histology and receptor studies.

### Results

The effects of various treatments on final tumour volume, body and tumour weights, and tumour doubling time in both experiments, are shown in Table I. At the end of the experiments, there were no significant differences in body weights between the groups.

In experiment I, all three bombesin/GRP antagonists significantly suppressed growth of SCLC H-69 tumours. RC-

**Table I** Effect of treatment with various peptide analogues on body and tumour weight, tumour volume and tumour doubling time in nude mice bearing xenografts of the human SCLC H69 and non-SCLC H157 cell lines

| *Treatment group* | *Tumour volume ($mm^3$)* *Initial* | *Final* | *Body weight (g)* | *Tumour weight (g)* | *Tumour doubling time (days)* |
|---|---|---|---|---|---|
| *Experiment I (SCLC-H69)* | | | | | |
| Control | 10.5 ± 1.6 | 249.7 ± 182.3 | 26.3 ± 2.3 | 0.27 ± 0.19 | 7.5 |
| RC-3440 | 10.0 ± 3.3 | 74.1 ± 84.1* | 26.2 ± 1.3 | 0.076 ± 0.04* | 12.1 |
| RC-3095 | 11.2 ± 1.8 | 80.2 ± 61.0* | 24.0 ± 2.4 | 0.081 ± 0.06* | 12.2 |
| RC-160 | 9.8 ± 1.8 | 66.0 ± 26.5* | 24.6 ± 1.2 | 0.058 ± 0.04* | 12.7 |
| RC-3950-II | 11.6 ± 4.1 | 49.0 ± 47.1* | 25.2 ± 1.7 | 0.03 ± 0.03* | 16.8 |
| *Experiment II (non-SCLC H157)* | | | | | |
| Control | 10.0 ± 2.0 | 1580.3 ± 455.7 | 26.5 ± 1.0 | 1.9 ± 0.3 | 3.88 |
| Castration | 9.6 ± 1.8 | 1326.7 ± 321.3 | 25.0 ± 2.2 | 1.35 ± 0.5 | 3.95 |
| RC-160 | 11.1 ± 2.6 | 291.0 ± 207.8* | 24.2 ± 4.7 | 0.64 ± 0.3* | 6.06 |
| RC-3095 | 11.2 ± 3.1 | 913.1 ± 412.3 | 25.1 ± 4.5 | 1.1 ± 0.6 | 4.42 |
| SB-75 | 9.7 ± 2.0 | 1301.0 ± 709.8 | 27.0 ± 2.0 | 1.4 ± 0.7 | 4.0 |

Values are means ± s.d. *$P<0.05$ vs control.

3440 and RC-3095 appeared to inhibit tumour growth to a similar extent. Therapy with bombesin/GRP antagonist RC-3950-II was the most effective and resulted in the greatest inhibition of tumour weight and volume (Figure 1a, Table I). Growth of SCLC H69 tumours in animals treated with the bombesin/GRP antagonists was significantly ($P<0.01$) inhibited within 14 days from start of the experiment. Tumour volume doubling time was prolonged by RC-3950-II treatment to 16.8 days, as compared with the control group, which has a doubling time of 7.5 days. Administration of somatostatin analogue RC-160 also significantly ($P<0.01$) inhibited tumour growth from day 14 until the end of the experiment (Figure 1a, Table I). The mean tumour weight was reduced significantly ($P<0.01$) by RC-160 compared with the control group (Table I). Tumour volume doubling time in mice receiving RC-160 was extended to 12.7 days (Table I).

In experiment II, only the therapy with somatostatin analogue RC-160 inhibited growth of non-SCLC H157 tumours (Figure 1b, Table I). The final tumour volume and tumour weight were significantly ($P<0.01$) reduced in animals receiving RC-160, compared with those of the controls (Table I). Tumour volume doubling time was prolonged by RC-160 to 6.06 days, as compared to 3.88 days for the control group. No significant reduction in final tumour volume, tumour weight and tumour growth could be found in the groups treated with bombesin/GRP antagonist RC-3095 or LH-RH antagonist SB-75. Castration also had no effect (Figure 1b).

Histologically, the SCLC H69 tumours were composed of uniform undifferentiated cells that were arranged in large solid nests. The highly cellular tumours contained very little stoma. The cells were elongated and the oval shaped and chromatin-rich nuclei were surrounded by narrow dark cytoplasm. Some of the tumour contained necrotic areas with inflammatory cell infiltration. The extent of necrosis was determined with a point counting method using a microscope ocular net. Mitotic and apoptotic indices were calculated and the data are shown in Table II. The necrosis was less extensive in the tumours treated with RC-3095 and RC-3950-II, but these differences from control were not significant statistically. The number of mitotic and apoptotic cells did not differ significantly from control data. However, the ratio of apoptotic to mitotic indices was significantly higher in the group receiving RC-160.

The non-SCLC H157 tumours consisted of large epithelial cells arranged in solid nests surrounded by very little stroma. The nuclei of tumour cells were pale, oval, slightly polymorphic, containing prominent nucleoli. Necrotic areas and a granulocytic infiltration could be seen in almost all tumours. There was no significant difference in the extent of necrosis among groups. The number of mitoses was not significantly changed by the treatments, but apoptosis was significantly enhanced after castration and especially after treatment with

**Figure 1** Tumour volumes in nude mice bearing (**a**) H69 human SCLC (O, control) during treatment with somatostatin analogue RC-160 (●), administered s.c. at a dose of 100 μg $day^{-1}$ per animal, and bombesin/GRP antagonists RC-3095 (Δ), RC-3440 (▲) and RC-3950-II (□), administered s.c. in doses of 20 μg $day^{-1}$ per animal, and (**b**) H157 human non-SCLC (O, control) during treatment with somatostatin analogue RC-160 (Δ), injected s.c. at a dose of 100 μg $day^{-1}$ per animal, bombesin/GRP antagonist RC-3095 (▲), administered s.c. at a dose of 20 μg $day^{-1}$ per animal, LH-RH antagonist SB-75 (□), given s.c. at a dose of 100 μg $day^{-1}$ per animal, and after castration ●). Vertical bars represent s.e. *$P<0.05$, **$P<0.01$ vs control.

SB-75. The ratio of apoptotic to mitotic indices was significantly higher only in the group treated with SB-75.

The levels of serum growth hormone (GH) and gastrin in control nude mice and in animals treated with peptide analogues in both experiments are shown in Table III. In both experiments, GH and gastrin levels in animals treated with RC-160 were significantly decreased compared with controls. There were no changes in levels of GH and gastrin after chronic treatment with bombesin/GRP antagonists or luteinising hormone-releasing hormone (LH-RH) antagonist SB-75 (Table III).

The characteristics of receptors for bombesin, somatostatin, EGF and IGF-I in H69 and H157 tumours were analysed following treatment with peptide analogues in both experiments, and the results are presented in Table IV. In experiment I, receptor assays on H69 tumour membranes showed high-affinity binding sites for bombesin/GRP, somatostatin, EGF and IGF-I. At the end of the experiment, concentration of receptors for bombesin/GRP was markedly decreased by treatment with bombesin/GRP antagonist RC-3440 and receptors were reduced to non-detectable levels by antagonists RC-3095 and RC-3950-II (Table IV). The binding capacity of EGF receptors was decreased after treatment with somatostatin analogue RC-160 or the bombesin/GRP antagonists. Therapy with RC-160 increased the binding capacity of receptors for somatostatin in membranes of H69 tumours (Table IV).

In experiment II, the results of receptor assays on membranes of non-SCLC H157 tumours demonstrated high-affinity binding sites for EGF and IGF-I, but the receptors for bombesin/GRP and somatostatin were absent (Table IV). A marked reduction in EGF binding capacity was observed after the treatment with RC-160, but not with bombesin/GRP antagonist RC-3095 or LH-RH antagonist SB-75. Somatostatin analogue RC-160 also decreased the binding capacity of IGF-I receptors in membranes of this tumour (Table IV). No changes in IGF-I binding capacity and affinity occurred after treatment with RC-3095, SB-75 or castration (Table IV).

In order to determine the specificity of the binding sites for EGF, IGF-I, bombesin/GRP and somatostatin on membranes of H-69 SCLC and H-157 non-SCLC, several structurally related and unrelated peptides such as GRP(14–27), [D-Trp[6]]LH-RH, somatostatin-14, hEGF and IGF-I were tested for their ability to inhibit binding of the radioligands. None of the peptides tested inhibited the binding of radiolabelled ligands at concentrations as high as 1 $\mu$M.

In studies *in vitro*, somatostatin analogue RC-160 added to the medium during the 5 days of incubation at concentrations of 0.001 to 10.0 $\mu g\ ml^{-1}$ significantly inhibited [$^3$H]thymidine incorporation into DNA of H69 SCLC cells (Table V). At 10.0 $\mu g\ ml^{-1}$ RC-160, DNA synthesis was suppressed by about 43%. In the presence of bombesin/GRP antagonists RC-3005 or RC-3009 in the medium at concentrations of 5.0 and 25.0 $\mu g\ ml^{-1}$ during the 3 days of incubation, [$^3$H]thymidine incorporation into the DNA of H69 cells (Table V) was also significantly suppressed.

**Table II** Effect of treatment with various peptide analogues and castration on mitotic and apoptotic indices in SCLC H69 and non-SCLC H157 tumours growing in nude mice

| *Groups* | *Mitotic index* | *Apoptotic index* | *Ratio of apoptotic to mitotic indices* |
|---|---|---|---|
| *Experiment I (SCLC H69)* | | | |
| Control | 37.9 ± 2.9 | 35.4 ± 2.6 | 0.96 ± 0.1 |
| RC-160 | 23.0 ± 4.6 | 42.3 ± 3.2 | 2.27 ± 0.5* |
| RC-3440 | 38.5 ± 4.8 | 32.5 ± 3.4 | 0.93 ± 0.2 |
| RC-3095 | 28.8 ± 3.8 | 36.5 ± 6.8 | 1.31 ± 0.3 |
| RC-3950-II | 49.8 ± 4.8 | 41.5 ± 1.5 | 0.85 ± 0.1 |
| *Experiment II (non-SCLC H157)* | | | |
| Control | 17.3 ± 4.0 | 2.87 ± 0.5 | 0.19 ± 0.04 |
| Castration | 24.2 ± 6.5 | 5.00 ± 0.5* | 0.29 ± 0.11 |
| RC-160 | 12.9 ± 1.2 | 4.00 ± 0.7 | 0.35 ± 0.08 |
| RC-3095 | 19.9 ± 2.4 | 4.36 ± 0.3 | 0.25 ± 0.04 |
| SB-75 | 11.8 ± 1.5 | 6.13 ± 0.8* | 0.57 ± 0.15* |

Values are means ± s.e. *$P < 0.05$ *vs* control.

**Table III** Serum gastrin and growth hormone (GH) levels in nude mice with xenografts of human SCLC-H69 and non-SCLC H157 cell lines after treatment with various peptide analogues

| *Treatment group* | *Gastrin (pg ml$^{-1}$)* | *Growth hormone (ng ml$^{-1}$)* |
|---|---|---|
| *Experiment I (SCLC H-69)* | | |
| Control | 130.0 ± 10.8 | 3.8 ± 0.5 |
| RC-160 | 68.0 ± 5.5** | 2.0 ± 0.5* |
| RC-3440 | 92.7 ± 8.2 | 3.5 ± 0.3 |
| RC-3095 | 100.7 ± 6.3 | 4.6 ± 0.5 |
| RC-3950-II | 128.2 ± 20.5 | 5.5 ± 1.2 |
| *Experiment II (non-SCLC H-157)* | | |
| Control | 113.7 ± 4.5 | 6.8 ± 0.8 |
| Castration | 98.9 ± 3.4 | 7.8 ± 2.5 |
| RC-160 | 57.5 ± 8.7** | 3.5 ± 0.3* |
| RC-3095 | 136.9 ± 12.4 | 8.0 ± 1.2 |
| SB-75 | 141.2 ± 13.8 | 5.0 ± 1.3 |

Values are means ± s.e. Values are mean ± s.e. *$P < 0.05$, **$P < 0.01$ *vs* control.

### Discussion

In the present study, we documented a significant growth-inhibitory effect of somatostatin analogue RC-160 (Octastatin) on the growth of the xenografts of human SCLC H69 cell line in nude mice. This effect was noted after 2 weeks of administration of RC-160 and persisted for the remaining treatment period of 3 weeks. Our results are in agreement with those previously reported by other groups, demonstrating inhibitory effects of different somatostatin analogues on the growth of SCLC cell lines, including H69, *in vivo* and *in vitro* (Bogden *et al.*, 1990; Taylor *et al.*, 1991). In our *in vitro* studies, we demonstrated that RC-160 significantly

**Table IV** Binding characteristics of EGF, IGF-I, bombesin/GRP and somatostatin receptors in membranes of SCLC H69 and non-SCLC H157 tumours after *in vivo* treatment with various peptide analogues

| | *EGF* | | *IGF-I* | | *Bombesin/GRP* | | *Somatostatin* | |
|---|---|---|---|---|---|---|---|---|
| *Groups* | $K_d$ (nM) | $B_{max}$ (fmol mg$^{-1}$ protein) | $K_d$ (nM) | $B_{max}$ (fmol mg$^{-1}$ protein) | $K_d$ (nM) | $B_{max}$ (fmol mg$^{-1}$ protein) | $K_d$ (nM) | $B_{max}$ (fmol mg$^{-1}$ protein) |
| *Experiment I (SCLC-H69)* | | | | | | | | |
| Control | 1.3 | 278 | 1.0 | 294 | 1.1 | 420 | 3.5 | 450 |
| RC-160 | 1.6 | 174 | 0.9 | 176 | 1.3 | 435 | 5.5 | 570 |
| RC-3440 | 1.0 | 134 | 0.7 | 255 | 0.9 | 255 | 5.0 | 501 |
| RC-3095 | 0.7 | 102 | 1.7 | 300 | ND | ND | 4.7 | 480 |
| RC-3950-II | 0.6 | 93 | 0.9 | 226 | ND | ND | 3.6 | 390 |
| *Experiment II (non-SCLC H157)* | | | | | | | | |
| Control | 0.7 | 249 | 0.5 | 257 | ND | ND | ND | ND |
| Castration | 0.6 | 210 | 0.6 | 233 | ND | ND | ND | ND |
| RC-3095 | 0.6 | 207 | 0.5 | 270 | ND | ND | ND | ND |
| RC-160 | 0.5 | 100 | 0.7 | 129 | ND | ND | ND | ND |
| SB-75 | 0.7 | 192 | 0.6 | 160 | ND | ND | ND | ND |

Binding characteristics were obtained from ten-point displacement experiments in triplicate tubes. No s.e. values provided because complete displacement assays on tumour membranes were done only once because of shortage of tumour material. ND, not detectable.

**Table V** Inhibitory effect of somatostatin analogue RC-160 and bombesin/GRP antagonists RC-3005 and RC-3009 on incorporation of [$^3$H]thymidine into DNA of SCLC H69 cells

| *Peptide analogues* | *Dose* $\mu g\ ml^{-1} day^{-1}$ | *Treatment time (days)* | *[$^3$H]Thymidine incorporation (per cent of control)* |
|---|---|---|---|
| *I Somatostatin analogue* | | 5 | |
| Control | | | 100.0 ± 3.5 |
| RC-160 | 0.001 | | 81.6 ± 4.6* |
| | 0.01 | | 80.0 ± 4.1* |
| | 0.1 | | 71.6 ± 3.4** |
| | 1.0 | | 73.3 ± 5.0** |
| | 10.0 | | 56.6 ± 12.0** |
| *II Bombesin GRP antagonists* | | 3 | |
| Control | | | 100.0 ± 1.5 |
| RC-3005 | 1.0 | | 100.0 ± 2.0 |
| | 5.0 | | 80.1 ± 1.5** |
| | 25.0 | | 81.6 ± 1.3** |
| RC-3009 | 1.0 | | 98.4 ± 0.8 |
| | 5.0 | | 92.7 ± 2.0* |
| | 25.0 | | 78.3 ± 2.6** |

[$^3$H]Thymidine (1–3 $\mu$Ci) was added 24 h before harvesting. Values are mean ± s.e. *$P<0.05$, **$P<0.01$ *vs* control.

inhibited tritiated thymidine incorporation into H-69 cells, indicating some direct effect of this analogue on tumour growth.

Antineoplastic actions of somatostatin analogues appear to involve multiple mechanisms. A significant fall in growth hormone (GH) levels induced by RC-160 could, through mechanisms involving suppression of endogenous growth factors such as IGF-I and IGF-II, be of major importance for the inhibition of tumour growth (Schally, 1988). Macauley *et al.* (1991) previously demonstrated that somatostatin analogue octreotide reduced IGF-I levels in patients with SCLC. Membrane receptors for IGF-I were demonstrated in human SCLC cell lines, and these cells could also be stimulated by IGF-I (Minuto *et al.*, 1988; Macaulay *et al.*, 1990). It was reported that immunoreactive IGF-I is detectable in primary and metastatic SCLC tumour tissue and in most SCLC cell lines (Macaulay *et al.*, 1988; Minuto *et al.*, 1988). In our study, serum GH levels in mice treated with RC-160 were decreased by about 48% as compared with control mice. The marked variation of serum GH levels between H-69 and H-157 control groups could be caused by different production and/or secretion of growth factors such as IGF-I by these two lung cancers. High levels of serum IGF-I might suppress the release of GH through a negative feedback on the hypothalamus or the anterior pituitary. In addition, since blood samples from animals bearing H-157 tumours were taken in the morning whereas those from mice with H-69 tumours were collected in the afternoon, the difference in GH levels between the two control groups might also be attributed to diurnal fluctuations of GH levels in those animals. Sinha *et al.* (1975) showed previously that serum levels of GH in two different strains of mice were usually high during the morning hours.

On the basis of our receptor assay results, which indicate the presence of high-affinity receptors for somatostatin on tumour membranes, analogues of somatostatin could also directly inhibit the growth of lung cancer cells. The inhibitory effect of somatostatin analogue RC-160 on [$^3$H]thymidine incorporation was shown on LNCaP prostatic cancer cells in culture (Gattani *et al.*, 1990). In the MIA PaCa-2 human pancreatic cancer cell line, somatostatin and its analogue RC-160 reversed the stimulatory effect of EGF on phosphorylation of the tyrosine kinase domain of the EGF receptors and on cell growth (Liebow *et al.*, 1989). These and other observations (Schally, 1988) suggest that somatostatin analogues can act as endogenous growth inhibitors in cancer cells through the activation of tyrosine phosphatase (Liebow *et al.*, 1989). Furthermore, somatostatin analogues may inhibit the secretion of bombesin-like peptides and the cAMP response to vasoactive intestinal peptide (Taylor *et al.*, 1991). The inhibitory effect of RC-160 on the growth of non-SCLC H157 tumours observed in our study is probably mainly due to suppression of GH and IGF-I secretion, since we did not find somatostatin receptors in membranes of this tumour. The absence of somatostatin receptors was also observed in tumour specimens obtained from patients with non-SCLC (Reubi *et al.*, 1990).

The present study demonstrates a significant inhibitory effect of bombesin/GRP antagonists RC-3095, RC-3440 and RC-3950-II on the growth of the SCLC H69 cell line xenografted into nude mice. In studies *in vitro*, we found that structurally related bombesin/GRP antagonists RC-3005 and RC-3009 significantly inhibited the incorporation of triatiated thymidine into DNA of H-69 cells, indicating that the inhibitory effects of this class of bombesin/GRP antagonists can be attributed at least in part to a direct action. RC-3095 and RC-3950-II also induced a reduction of bombesin/GRP receptors to non-detectable levels in membranes of this tumour. Previous studies have shown that bombesin and GRP are secreted from SCLC cells into tissue culture medium and that high-affinity receptors for bombesin/GRP are present in several SCLC cell lines including H69 (Layton *et al.*, 1988; Mahmoud *et al.*, 1991; Thomas *et al.*, 1992; Moody & Cuttitta, 1993). Since bombesin stimulates the clonal growth of SCLC and DNA synthesis *in vitro* (Carney *et al.*, 1987) and the growth of SCLC xenografts in nude mice (Alexander *et al.*, 1988), the inhibition of H69 tumour growth by bombesin/GRP antagonists appears to be brought about by blockade of bombesin/GRP receptors on H69 cells.

Previously, we have shown that inhibition of growth of various cancers, including pancreatic, prostatic, mammary and colorectal by antagonist RC-3095, was associated with a major decrease in EGF receptor levels in tumour membranes (Radulovic *et al.*, 1991*b*; Szepeshazi *et al.*, 1991,1992; Pinski *et al.*, 1994*a*, *b*). Thus, bombesin/GRP antagonists may act locally by various mechanisms which result in a reduction in the available binding sites for EGF. Most non-SCLC and SCLC cell lines express the EGF receptor (Veale *et al.*, 1987; Tateishi *et al.*, 1991; Damstrup *et al.*, 1992; Rabiasz *et al.*, 1992).

The exact molecular mechanism of action of bombesin/GRP antagonists on EGF receptors is still not well understood. Bombesin initiates a series of intracellular signals, which cause an increase in inositol 1,4,5-triphosphate, a mobilisation of $Ca^{2+}$ and diacylglycerol production, leading to activation of protein kinase C (Zachary *et al.*, 1986; Langdon *et al.*, 1992; Szepeshazi *et al.*, 1992). Activation of protein kinase C causes phosphorylation of EGF receptors on threonine residues. Bombesin and GRP have been shown to enhance the phosphorylation of EGF receptors, and antagonist RC-3095 inhibits these effects in various cancer lines and cancer specimens (Liebow *et al.*, 1992). These results suggest that bombesin and GRP may function by upregulating EGF receptors and that antagonist RC-3095 prevents this up-regulation (Liebow *et al.*, 1992). Bombesin/GRP antagonists may also block early cellular events that precede calcium mobilisation and stimulation of mitogenesis (Woll & Rozengurt, 1988).

In contrast to its inhibitory effects on SCLC, bombesin/GRP antagonist RC-3095 did not affect the growth of H157 non-SCLC. Our observations can be explained by the absence of bombesin/GRP receptors in membranes of this tumour and are supported by the previously reported finding that non-SCLC cell lines do not express detectable levels of bombesin-like peptides (Cuttitta *et al.*, 1985). A study on various SCLC and non-SCLC cell lines demonstrated that the GRP gene is expressed in four of six classic SCLC cell lines, but not in variant SCLC and non-SCLC cell lines (Cardona *et al.*, 1991).

Since sex hormone receptors have been reported in human lung tumours (Beattie *et al.*, 1985; Cagle *et al.*, 1990) and the incidence of pulmonary neoplasms is influenced by sex hormones in laboratory animals (Noronha & Goodhall *et al.*, 1983), we felt that it was important to determine whether castration or administration of LH-RH antagonist SB-75 could inhibit the growth of H157 tumours. However, despite an increased apoptosis in tumours from castrated or SB-75-treated animals, no difference in tumour volumes and weights was found compared with controls. Thus, it appears that growth factors such as IGF-I play a more important role than sex hormones in the stimulation of H157 cells.

In view of the heterogeneity of lung cancers, i.e. non-SCLC is subclassified into squamous cell carcinoma, adenocarcinoma and large cell carcinoma, and SCLC into a classic and a variant subclass, it is difficult to make general conclusions about the utility of our peptide analogues for the treatment by studying single examples of two subclasses of lung cancer. Nevertheless, our findings confirm the view, which is based on a large number of other studies, that somatostatin analogues such as RC-160 and bombesin/GRP antagonists such as RC-3095, RC-3440 and RC-3950-II could be considered as potentially useful agents for treatment of SCLC. Significant variations in binding sites for these compounds between H-69 SCLC and H-157 non-SCLC xenografts raise the possibility that such analogues could be used more selectively in the treatment of various subtypes of lung cancer. Our work supports the merit of further investigations based on these and other analogues of somatostatin and bombesin/GRP antagonists.

**Abbreviations:** LH-RH, luteinising hormone-releasing hormone; GH, growth hormone; EGF, epidermal growth factor; IGF-I, insulin-like growth factor I or somatomedin C; TGF-α, transforming growth factor α; GRP, gastrin-releasing peptide; SCLC, small-cell lung carcinoma; non-SCLC, non-small cell lung carcinoma; Tpi,2,3,4,9-tetrahydro-1H-pyrido[3,4-*b*]-3-carboxylic acid; HPLC, high-performance liquid chromatography; cAMP, cyclic adenosine monophosphate; Tac, thiazolidine-4-carboxylic acid.

The studies on the effects of RC-160 and bombesin/GRP antagonists on SCLC cell line H69 *in vitro* were carried out by Tommie W. Redding, to whom we express our thanks. We are grateful to ASTA Medica (Frankfurt/Main, Germany) for SB-75 (Cetrorelix) and RC-3095 and Debiopharm (Lausanne, Switzerland) for RC-160. We thank the National Hormone and Pituitary Program of the National Institute of Diabetes and Digestive and Kidney Diseases for the gifts of materials used in radioimmunoassays. We are grateful to Dr H. Oie (NCI-Navy Medical Oncology Branch) for providing NCI-H157 non-SCLC line. This work was supported by the National Institute of Health Grant CA 40077 and by the Medical Research Service of the Veterans Affairs (to A.V.S.). The contents of this manuscript are solely the responsibility of the authors and do not necessarily represent the official view of the National Cancer Institute.

**References**

ALEXANDER, R.W., UPP, Jr, J.R., POSTON, G.J., GUPTA, V., TOWNSEND, Jr, C.M. & THOMPSON, J.C. (1988). Effects of bombesin on growth of human small cell lung carcinoma *in vivo*. *Cancer Res.*, **48**, 1439–1441.

BAJUSZ, S., CSERNUS, V.J., JANAKY, T., BOKSER, L., FEKETE, M. & SCHALLY, A.V. (1988). New antagonists of LHRH: II. Inhibition and potentiation of LHRH by closely related analogues. *Int. J. Peptide. Protein. Res.*, **32**, 425–435.

BEATTIE, C.W., HANSEN, N.W. & THOMAS, P.A. (1985). Steroid receptors in human lung cancer. *Cancer Res.*, **45**, 4206–4214.

BOGDEN, A.E., TAYLOR, J.E., MOREAU, J.P., COY, D.H. & LEPAGE, D.J. (1990). Response of human lung tumour xenografts to treatment with a somatostatin analogue (Somatuline). *Cancer Res.*, **50**, 4360–4365.

BORING, C.C., SQUIRES, T.S. & TONG, T. (1992). Cancer statistics, 1992. *Ca-A Cancer J. Clin.*, **42**, 19–38.

CAGLE, P.T., MODY, D.R. & SCHWARTZ, M.R. (1990). Estrogen and progesterone receptors in brochogenic carcinoma. *Cancer Res.*, **50**, 6632–6635.

CAI, R.Z., SZOKE, B., LU, R., FU, D., REDDING, T.W. & SCHALLY, A.V. (1986). Synthesis and biological activity of highly potent octapeptide analogs of somatostatin. *Proc. Natl Acad. Sci. USA*, **83**, 1896–1900.

CAI, R.Z., RADULOVIC, S., PINSKI, J., NAGY, A., REDDING, T.W., OLSEN, D.B. & SCHALLY, A.V. (1992). Pseudononapeptide bombesin antagonists containing C-terminal Trp or Tpi. *Peptides*, **13**, 267–271.

CAI, R.-Z., REILE, H., ARMATIS, P. & SCHALLY, A.V. (1994). Potent bombesin antagonists with a $Leu^{13}$ $\psi(CH_2N)Tac^{14}$C-terminal. *Proc. Natl Acad. Sci. USA* (in press).

CARDONA, C., RABBITTS, P.H., SPINDEL, E.R., GHATEI, M.A., BLEEHEN, N.M., BLOOM, S.R. & REEVE, J.G. (1991). Production of neuromedin B and neuromedin B gene expression in human lung tumor cell lines. *Cancer Res.*, **51**, 5205–5211.

CARNEY, D.N., CUTTITTA, F., MOODY, T.W. & MINNA, J.D. (1987). Selective stimulation of small cell lung cancer clonal growth by bombesin and gastrin releasing peptide. *Cancer Res.*, **47**, 821–825.

CUTTITTA, F., CARNEY, D.N., MULSHINE, J.W., MOODY, T.W., FEDORKO, J., FISCHLER, A. & MINNA, J.D. (1985). Bombesin-like peptides can function as autocrine growth factors in human small cell lung cancer. *Nature*, **316**, 823–826.

DAMSTRUP, L., RYGAARD, K., SPANG-THOMSEN, M., POULSON, H.S. (1992). Expression of the epidermal growth factor receptor in human small cell lung cancer cell lines. *Cancer Res.*, **52**, 3089–3093.

GATTANI, A., BROWER, S., PLATICA, M., SCHALLY, A.V. & HOLLANDER, V. (1990). The inhibition of thymidine incorporation by LHRH and somatostatin analogs on prostatic cancer cell line LNCaP in culture. *Proc. Am. Assoc. Cancer Res.*, **1299**, 219.

HALMOS, G., REKASI, Z., SZOKE, B. & SCHALLY, A.V. (1993). Use of radioreceptor assay and cell superfusion system for *in vitro* screening of analogs of growth hormone-releasing hormome. *Receptor*, **3**, 87–97.

LANGDON, S., SEITHI, T., RITCHIE, A., MUIR, M., SMYTHE, J. & ROZENGURT, E. (1992). Broad spectrum neuropeptide antagonists inhibit the growth of small cell lung cancer *in vivo*. *Cancer Res.*, **52**, 4554–4557.

LAYTON, J.E., SCANLON, D.B., SOVENY, C. & MORSTYN, G. (1988). Effects of bombesin antagonists on the growth of small cell lung cancer cells *in vitro*. *Cancer Res.*, **48**, 4783–4789.

LIEBOW, C., REILLY, C., SERRANO, M. & SCHALLY, A.V. (1989). Somatostatin analogs inhibit growth of pancreatic cancer by stimulating tyrosine phosphatase. *Proc. Natl Acad. Sci. USA*, **86**, 2003–2007.

LIEBOW, C., LEE, M.T., KREBS, L.J. & SCHALLY, A.V. (1992). Bombesin may stimulate growth through up-regulation of EGF receptors. *Pancreas*, **7**, 746.

MACAULAY, V.M., TEALE, J.D., EVERARD, M.J., JOSHI, G.P., SMITH, I.E. & MILLAR, J.L. (1988). Somatomedin-C/insulin-like growth factor-I is a mitogen for human small cell lung cancer. *Br. J. Cancer*, **57**, 91–93.

MACAULAY, V.M., EVERARD, M.J., TEALE, D., TROUTT, P.A., VAN WYK, J.J., SMITH, I.E. & MILLAR, J.L. (1990). Autocrine function of insulin-like growth factor I in human small cell lung cancer cell lines and fresh tumor cells. *Cancer Res.*, **50**, 2511–2517.

MACAULAY, V.M., SMITH, I.E., EVERARD, M.J., TEALE, J.D., REUBI, J.-C. & MILLAR, J.L. (1991). Experimental and clinical studies with somatostatin analogue octreotide in small cell lung cancer. *Br. J. Cancer*, **64**, 451-456.

MAHMOUD, S., STALEY, J., TAYLOR, J., BODGEN, A., MOREAU, J.-P., COY, D., AVIS, I., CUTTITTA, F., MULSHINE, J.L. & MOODY, T.W. (1991). [Psi$^{13,14}$] Bombesin analogues inhibit growth of small cell lung cancer *in vitro* and *in vivo*. *Cancer Res.*, **51**, 1798-1802.

MINUTO, F., DEL MONTE, P., BARRAECA, A., ALAMA, A., CARIOLA, G. & GIORDANO, G. (1988). Evidence for autocrine mitogenic stimulation by somatomedin-C/insulin-like growth factor I on an established human lung cancer cell line. *Cancer Res.*, **48**, 3716-3719.

MOODY, T.W. & CUTTITTA, F. (1993). Growth factor and peptide receptors in small cell lung cancers. *Life Sci.*, **52**, 1161-1173.

MUNSON, P.J. & RODBARD, D. (1980). LIGAND: a versatile computerized approach for characterization of ligand-binding systems. *Anal. Biochem.*, **107**, 220-239.

NORONHA, R.F.X. & GOODALL, C.M. (1983). Enhancement of testosterone of dimethylnitrosamine carcinogenesis in lung, liver and kidney of inbred NZR/Gd female rats. *Carcinogenesis*, **4**, 613-616.

PINSKI, J., REILE, H., HALMOS, G., GROOT, K. & SCHALLY, A.V. (1994*a*). Inhibitory effects of somatostatin analogue RC-160 and bombesin/gastrin-releasing peptide antagonist RC-3095 on the growth of the androgen-independent Dunning R-3327-AT-1 rat prostate cancer. *Cancer Res.*, **54**, 169-174.

PINSKI, J., HALMOS, G., YANO, T., SZEPESHAZI, K., QIN, Y., ERTL, T. & SCHALLY, A.V. (1994*b*). Inhibition of growth of MKN45 human gastric adenocarcinoma xenografts in nude mice by treatment with bombesin/gastrin releasing peptide antagonist (RC-3095), and somatostatin analogue RC-160. *Int. J. Cancer*, **57**, 574-580.

RABIASZ, G.J., LANGDON, S.P., BARTLETT, J.M.S., CREW, A.J., MILLER, E.P., SCOTT, W.N., SMYTH, J.F. & MILLER, W.R. (1992). Growth control by epidermal growth factor and transforming growth factor-α in human lung squamous carcinoma cells. *Br. J. Cancer*, **66**, 254-259.

RADULOVIC, S., CAI, R.-Z., SERFOZO, P., GROOT, K., REDDING, T.W., PINSKI, J. & SCHALLY, A.V. (1991*a*). Biological effects and receptor binding affinities of new pseudonanapeptide bombesin/GRP receptor antagonists with N-terminal D-Trp or D-Tpi. *Int. J. Peptide Protein Res.*, **38**, 593-600.

RADULOVIC, S., MILLER, G. & SCHALLY, A.V. (1991*b*). Inhibition of growth of HT-29 human colon cancer xenografts in nude mice by treatment with bombesin/gastrin releasing peptide antagonist (RC-3095). *Cancer Res.*, **51**, 6006-6009.

REUBI, J.C., WASER, B., SHEPPARD, M. & MACAULAY, V. (1990). Somatostatin receptors are present in small-cell but not in non-small-cell primary lung carcinomas: Relationship to EGR-receptors. *Int. J. Cancer*, **45**, 269-274.

SCHALLY, A.V. (1988). Oncological application of somatostatin analogues. *Cancer Res.*, **48**, 6977-6985.

SETHI, T. & ROZENGURT, E. (1992). Multiple neuropeptides stimulate clonal growth of small cell lung cancer: effects of bradykinin, vasopressin, cholecystokinin, galanin, and neurotensin. *Cancer Res.*, **51**, 3621-3623.

SIEGFRIED, J.M. & OWENS, S.E. (1988). Response of primary human lung carcinomas to autocrine growth factors produced by a lung carcinoma cell line. *Cancer Res.*, **48**, 4076-4981.

SINHA, Y.N., SALOCKS, C.B., VANDERLAAN, W.P. (1975). Prolactin and growth hormone levels in different inbred strains of mice: patterns in association with estrous cycle, time of day, and perphenazine stimulation. *Endocrinology*, **97**, 1112-1121.

SONDAK, V.K., BERTELSEN, C.A., TANIGAWA, N., HILDEBRAND-ZANKI, S.U., MORTON, D.L., KORN, E.L. & KERN, D.H. (1984). Clinical correlations with chemosensitivities measured in a rapid thymidine incorporation assay. *Cancer Res.*, **44**, 1725-1728.

STALEY, J., COY, D., TAYLOR, J.E., KIM, S. & MOODY, T.W. (1991). [Des-Met$^{14}$]Bombesin analogues function as small cell lung cancer bombesin receptor antagonists. *Peptides*, **12**, 145-149.

STEEL, R.G.D. & TORRIE, J. (1976). Principles and procedures of statistic 114, p. 114. McGraw-Hill: New York.

SRKALOVIC, G., SZENDE, B., REDDING, T.W., GROOT, K. & SCHALLY, A.V. (1989). Receptors for D-Trp-6-luteinizing hormone-releasing hormone, somatostatin and insulin-like growth factor I in MXT mouse mammary carcinoma. *Proc. Soc. Exp. Biol. Med.*, **192**, 209-218.

SZEPESHAZI, K., SCHALLY, A.V., CAI, R.-Z., RADULOVIC, S., MILOVANOVIC, S. & SZOKE, B. (1991). Inhibitory effect of bombesin/GRP antagonist RC-3095 and high dose of somatostatin analog RC-160 on nitrosamine-induced pancreatic cancers in hamsters. *Cancer Res.*, **51**, 5980-5986.

SZEPESHAZI, K., SCHALLY, A.V., HALMOS, G., GROOT, K. & RADULOVIC, S. (1992). Antagonist of bombesin/Gastrin releasing peptide inhibits growth of estrogen dependent and independent MXT mammary cancers in mice. *J. Natl Cancer Inst.*, **84**, 1915-1921.

TATEISHI, M., ISHIDA, T., MITSUDOMI, T. & SUGIMACHI, K. (1991). Prognostic implication of transforming growth factor α in adenocarcinoma of the lung – an immunohistochemical study. *Br. J. Cancer*, **63**, 130-133.

TAYLOR, J.E., MOREAU, J.P., BAPTISTE, L. & MOODY, T.W. (1991). Octapeptide analogues of somatostatin inhibit the clonal growth and vasoactive intestinal peptide-stimulated cyclic AMP formation in human small cell lung cancer cells. *Peptides*, **12**, 839-843.

THOMAS, F., ARVELO, F., ANTOINE, E., JACROT, M. & POUPON, M.F. (1992). Antitumor activity of bombesin analogues on small cell lung cancer xenografts: relationship with bombesin receptor expression. *Cancer Res.*, **52**, 4872-4877.

VEALE, S., ASHCROFT, T., MARCH, C., GIBSON, G.J. & HARRIS, A.L. (1987). Epidermal growth factor receptors in non-small cell lung cancer. *Br. J. Cancer*, **55**, 513-516.

WOLL, P.J. & ROZENGURT, E. (1988). [D-Arg$^{1}$, D-Phe$^{5}$, D-Trp$^{7,9}$, Leu$^{11}$]substance P, a potent bombesin antagonist in murine Swiss 3T3 cells, inhibits the growth of human small cell lung cancer cells *in vitro*. *Proc. Natl Acad. Sci. USA*, **85**, 1859-1863.

ZACHARY, I., SINNETT-SMITH, J.W., ROZENGURT, E. (1986). Early events elicted by bombesin and structurally related peptides in quienscent Swiss 3T3 cells. I. Activation of protein kinase C and inhibition of epidermal growth factor binding. *J. Cell Biol.*, **102**, 2111-2222.